# Neutrophil Activation: Influence of Antimony Tolerant and Susceptible Clinical Strains of *L. (V.) panamensis* and Meglumine Antimoniate

**DOI:** 10.3389/fcimb.2021.710006

**Published:** 2021-09-22

**Authors:** Olga Lucía Fernández, Lady Giovanna Ramírez, Míriam Díaz-Varela, Fabienne Tacchini-Cottier, Nancy Gore Saravia

**Affiliations:** ^1^Centro Internacional de Entrenamiento e Investigaciones Médicas (CIDEIM), Cali, Colombia; ^2^Universidad Icesi, Cali, Colombia; ^3^Department of Biochemistry, University of Lausanne, Epalinges, Switzerland

**Keywords:** neutrophil activation, *Leishmania*, meglumine antimoniate, antileishmanial drug susceptibility, intrinsic drug resistance

## Abstract

Emerging evidence indicates that innate host response contributes to the therapeutic effect of antimicrobial medications. Recent studies have shown that *Leishmania* parasites derived by *in vitro* selection for resistance to pentavalent antimony (SbV) as meglumine antimoniate (MA) modulate the activation of neutrophils. However, whether modulation of neutrophil activation extends to natural resistance to this antileishmanial drug has not been established. We have evaluated the influence of clinical strains of *L. (V.) panamensis* having intrinsic tolerance/resistance to SbV, on the inflammatory response of neutrophils during *ex vivo* exposure to MA. Accordingly, neutrophils obtained from healthy donors were infected with clinical strains that are sensitive (n = 10) or intrinsically tolerant/resistant to SbV (n = 10) and exposed to a concentration approximating the maximal plasma concentration (Cmax) of SbV (32 µg/ml). The activation profile of neutrophils was evaluated as the expression of the surface membrane markers CD66b, CD18, and CD62L by flow cytometry, measurement of reactive oxygen species (ROS) by luminometry, and NET formation using Picogreen to measure dsDNA release and quantification of NETs by confocal microscopy. These parameters of activation were analyzed in relation with parasite susceptibility to SbV and exposure to MA. Here, we show that clinical strains presenting intrinsic tolerance/resistance to SbV induced significantly lower ROS production compared to drug-sensitive clinical strains, both in the presence and in the absence of MA. Likewise, analyses of surface membrane activation markers revealed significantly higher expression of CD62L on cells infected with intrinsically SbV tolerant/resistant *L. (V.) panamensis* than cells infected with drug-sensitive strains. Expression of other activation markers (CD18 and CD66b) and NET formation were similar for neutrophils infected with SbV sensitive and tolerant clinical strains under the conditions evaluated. Exposure to MA broadly impacted the activation of neutrophils, diminishing NET formation and the expression of CD62L, while augmenting ROS production and CD66b expression, independently of the parasite susceptibility phenotype. These results demonstrated that activation of human neutrophils *ex vivo* is differentially modulated by infection with clinical strains of *L. (V.) panamensis* having intrinsic tolerance/resistance to SbV compared to sensitive strains, and by exposure to antimonial drug.

## Introduction

Cutaneous leishmaniasis (CL) is a neglected tropical disease of emerging or reemerging public health importance in 98 countries, with more than a million new cases reported annually (Alvar et al., 2012; Pace, 2014). First-line treatment in Latin America is parentally administered meglumine antimoniate (MA), and orally administered miltefosine (ML) is recommended for individuals with contraindication for antimonial drugs. Case identification and treatment are the principal control measures for CL; however, treatment failure within efficacy trials is frequent (17 to 47%) (Palacios et al., 2001; Soto et al., 2008; Velez et al., 2010; Chrusciak-Talhari et al., 2011; Rubiano et al., 2012). The determinants of antileishmanial treatment failures are multiple, complex, and poorly understood; however, it is clear that both parasite and host factors including innate and adaptive immune response contribute to therapeutic outcome (Rojas et al., 2006; Goyeneche-Patino et al., 2008; Obonaga et al., 2014; Palic et al., 2019).

Evidence of parasite resistance to SbV and MIL has been reported globally from major endemic regions. Differences in susceptibility to SbV were found among clinical strains of *L. (V.) panamensis* circulating in Colombia that are distinguished by isoenzyme profiles that define discrete natural parasite populations discernable as zymodemes (Saravia et al., 1998). Subpopulations of *L. (V.) panamensis* belonging to zymodeme 2.3 were subsequently found to be associated with tolerance/resistance to SbV, while those belonging to zymodeme 2.2 were associated with sensitivity (Fernandez et al., 2014). The relationship between drug susceptibility of *Leishmania* as conventionally measured *in vitro* and therapeutic response remains unclear for all forms of human leishmaniasis. The multifactorial bases of the response to treatment confound the understanding of parasite drug susceptibility as a determinant of therapeutic outcome. However, the importance of the host cell in determining susceptibility is illustrated by the distinct EC_50_ of antileishmanial drugs for the same parasite in different host cells (primary macrophages or mononuclear phagocytic cell lines (Seifert et al., 2010)), and the divergence of drug screening results based on intracellular amastigotes, promastigotes, and axenic amastigotes (Vermeersch et al., 2009; De Muylder et al., 2011; Zahid et al., 2019). Furthermore, growing evidence indicates that neutrophils, the first host cells recruited to the site of infection, may play a role in the pathogenesis of leishmaniasis depending on the infecting *Leishmania* spp (Afonso et al., 2008; Carlsen et al., 2013; Hurrell et al., 2015; Charmoy et al., 2016; Hurrell et al., 2016; Passelli et al., 2021). Despite their important role in cutaneous leishmaniasis, the involvement of neutrophils in the response to antileishmanial drugs has not been examined.

Neutrophils are the most abundant cells in human blood, are terminally differentiated cells, and are rapidly recruited to sites of infection. Their antimicrobial mechanisms include phagocytosis, the formation of radical oxygen species (ROS), and the release of neutrophil extracellular traps (NETs) (Brinkmann et al., 2004) as well as antimicrobial agents stored in neutrophil granules that can be rapidly released into the phagosomes (Borregaard, 2010). Previous studies have shown that elimination or survival of *Leishmania* parasites by or within neutrophils differs among parasite species or even strains of the same species (Hurrell et al., 2016; Regli et al., 2017). *Leishmania* may be killed or inhibit the otherwise very efficient killing machinery of these cells (Passelli et al., 2021) and effectively mediate the transfer of parasites to macrophages (van Zandbergen et al., 2004; Chaves et al., 2020). Infection of human neutrophils with laboratory-derived MIL and SbV resistant lines of *L. (V.) panamensis* elicited significantly greater NET formation by both murine and human neutrophils compared to infections with the wild-type (WT) sensitive line, while the MIL resistant line, but not SbV resistant line, also elicited significantly higher ROS production than SbV resistant or sensitive lines (Regli et al., 2018). The potential of naturally resistant *Leishmania* populations to modulate neutrophil activation has not been previously evaluated and is relevant to treatment since mechanisms of resistance derived by *in vitro* drug selection have not replicated intrinsic or acquired resistance mechanisms evidenced during treatment or *in vitro* evaluation of drug susceptibility (Goyeneche-Patino et al., 2008).

In this study, we have evaluated the influence of clinical strains of having intrinsic tolerance/resistance to SbV on the inflammatory response of neutrophils during *ex vivo* infection and treatment with MA.

## Materials and Methods

### Study Design

Human neutrophils were infected *ex vivo* with SbV sensitive (n = 10) or resistant (n = 10) clinical strains of *L. (V.) panamensis*. Neutrophil effector functions were assessed by analyzing the production of reactive oxygen species (ROS), the formation of neutrophil extracellular traps (NETs), and the expression of cell surface activation markers (CD18, CD62L, and CD66b). Susceptibility phenotype of clinical strains as intracellular amastigotes was previously evaluated *in vitro* as described (Fernandez et al., 2014) and confirmed within the scope of this study using macrophages differentiated from U937 human promonocytic cells.

### Cell Donors

To characterize the phenotype and functionality of human neutrophils during infection with *L. (V.) panamensis* strains and their relation with parasite drug susceptibility, 13 volunteer healthy donors without history of leishmaniasis were recruited to participate in this study. The inclusion criteria were as follows: 18–60 years of age, absence of active or healed lesions suggestive of leishmaniasis, voluntary participation, and hemoglobin levels > 11 mg/dl in women and >12 mg/dl in men.

### Ethics Statement

The institutional ethical review board of Centro Internacional de Entrenamiento e Investigaciones Médicas (CIDEIM) approved all study procedures in accordance with national guidelines: CEIH approval code: 1274, and was conducted in compliance with the legislation of the Canton of Vaud and the Swiss Confederation (CER-VD 2017-00182) and in accordance with the international guidelines: WMA Helsinki Declaration, 2013 declaration of Helsinki. Written informed consent was obtained from all donors who participated voluntarily in the study.

### *Leishmania (Viannia) panamensis* Internal Reference Lines and Clinical Strains

Internal controls included a wild-type (WT) antimony-sensitive line of *L. (V.) panamensis* transfected with the luciferase reporter gene (luc), MHOM/COL/03/1166, prepared as previously described (Roy G, Dumas 2000), and antimony resistant laboratory line of *L. (V.) panamensis* (MHOM/COL/03/1166-1000.1). Clinical strains of *L. (V.) panamensis* previously defined as sensitive or resistant to antimony were obtained from CIDEIM Biobank. Susceptibility to the C_max_ of SbV, zymodeme, clinical outcome of treatment with MA of the corresponding patient, and geographic origin of strains included in the study are summarized in **Table 1**. *Leishmania* strains were propagated in culture at 25°C in Senejke’s diphasic blood agar medium with 5 ml of PBS for 6 days. Parasites were opsonized in 10% human AB serum for 60 min 34°C prior to exposure to neutrophils.

**Table 1:** Characteristics of clinical strains of *L. (V.) panamensis*.

Strain code	Zymodeme	% Reduction of parasite burden(32 µg SbV/mL)		Treatment outcome	Department
		Historical data^a^	Confirmation of susceptibility		
MHOM/CO/2014/12309	2.2	99	80	Cure	Nariño
MHOM/CO/2015/12345	2.2	99	89	Cure	Nariño
MHOM/CO/2011/10763	2.2	99	75	Failure	Nariño
MHOM/CO/2014/11126	2.2	97	92	Failure	Nariño
MHOM/CO/2015/12367	2.2	97	85	Cure	Valle
MHOM/CO/2013/11006	2.2	99	91	Failure	Nariño
MHOM/CO/2014/11075	2.2	99	97	Failure	Nariño
MHOM/CO/2013/11031	2.2	98	97	Failure	Nariño
MHOM/CO/2013/10977	2.2	99	97	Cure	Nariño
MHOM/CO/2014/11109	2.2	99	83	Cure	Nariño
MHOM/CO/2015/12355	2.3	45	45	Cure	Nariño
MHOM/CO/2014/12251	2.3	46	48	Cure	Valle
MHOM/CO/2011/10772	2.3	14	45	Failure	Nariño
MHOM/CO/2013/11045	2.3	45	43	Failure	Nariño
MHOM/CO/1984/2169	2.3	0	28	Failure	Nariño
MHOM/CO/2013/12116	2.3	49	24	Failure	Chocó
MHOM/CO/2013/11024	2.3	45	58	Failure	Nariño
MHOM/CO/2013/11026	2.3	46	60	Failure	Nariño
MHOM/CO/2014/11152	2.3	56	26	Cure	Nariño
MHOM/CO/2013/7158	2.3	70	33	Cure	Chocó

^a^Historical data correspond to previously determined drug susceptibility, and confirmation refers to the result obtained within the scope of this study.

### *In Vitro* Assay for SbV Susceptibility Using Intracellular Amastigotes

The *in vitro* susceptibility of clinical strains of *L. (V.) panamensis* was determined as intracellular amastigotes. Susceptibility was based on the percent reduction of intracellular parasite burden in U-937 macrophages (ATCC CRL-159.3) by 72 h of exposure to 32 µg Sb^V^/ml as meglumine antimoniate as previously described (Fernandez et al., 2012). Briefly, 1.2 x 10^5^ U-937 cells cultured in 24-well plates containing glass coverslips were differentiated to macrophages by treatment with phorbol 12-myristate 13-acetate (PMA; 100 ng/ml; Sigma) and infected with opsonized promastigotes at a ratio of 5 parasites per macrophage. After 2 h of co-culture at 34°C in 5% CO_2_, extracellular parasites were removed by washing with PBS, and infected cells were cultured in RPMI-1640 medium (Sigma-Aldrich) supplemented with 10% heat-inactivated fetal bovine serum (FBS; 10082; Gibco), 1% Penicillin/Streptomycin solution (10,000 U/ml Penicillin G/10,000 μg/ml Streptomycin; Gibco BRL), and 1% glutamine during 24 h to allow differentiation of intracellular parasites to amastigotes. The medium was replaced with complete RPMI containing 32 µg Sb^V^/ml and replenished 48 h later, followed by incubation for an additional 24 h.

Glass coverslips with infected cells were fixed with methanol and stained with 3% Giemsa (Sigma Aldrich) prior to blinded evaluation by one of the two experienced microscopists who evaluated all of the slides for this study. Six replicates of cells exposed to the discriminatory drug concentrations and infected control macrophages not exposed to the drugs were evaluated. The number of intracellular amastigotes per cell was determined for 100 macrophages per replica. Susceptibility results were expressed as percent reduction of infection, determined by comparing the parasite burden of infected cells exposed to SbV *versus* the parasite burden of infected cells cultured without drug.

### Human Neutrophil Isolation

Peripheral blood neutrophils were isolated from the venous blood of healthy volunteers. Blood volume ranged from 20 to 200 ml according to the experimental protocol. Density gradient centrifugation using polymorphprep (Progen) was performed, and neutrophils were isolated according to the manufacturer’s instructions.

### NET Detection

NET formation was first assessed by the measurement of dsDNA in culture supernatants using the QuantiT PicoGreen kit (Thermo Fisher) as previously described (Regli et al., 2018). Briefly, for each condition, 2 million (2 × 10^6^/500 µl) neutrophils were primed with GM-CSF (25 ng/ml) for 20 min at 37°C. The neutrophils were incubated for 4 h in the *ex vivo* medium (Lonza Bioscience) with (100 ng/ml) PMA, *L. (V.) panamensis* strains, or without stimulus at 34°C, with rotation. After incubation, DNAse (2.5 UI/ml) was added to the cell suspensions. The reaction was stopped using EDTA (2.5 mM). The supernatants were collected and transferred to black 96-well plates. Picogreen dye was added, and fluorescence was measured using a plate reader (Chamaleon) at an excitation wavelength of 480 nm and an emission wavelength of 520 nm.

NET release and quantification was also assessed by confocal microscopy. Human neutrophils (2.5 × 10^5^ per well) were seeded on poly-L-lysine (Sigma) coated microscope slides (Thermo Scientific) in RPMI 1640 (Gibco) supplemented with 2% heat-inactivated AB human serum (Sigma). Neutrophils were exposed for 4 h to either *L. (V.) p*. at a parasite/cell ratio of 5:1 in the presence or absence of 32 µg SbV per ml during the last 2 h of infection. In parallel, cells were exposed to 100 ng/ml PMA, 100 ng/ml PMA plus 100 ng/ml DNase I, or the culture medium alone. After incubation, cells were fixed with 4% paraformaldehyde, washed and blocked with PBS containing 3% BSA. Cells were incubated with goat antihuman MPO primary antibody (R&D, AF3667), extensively washed, and incubated with Alexa-Fluor 488 anti-goat IgG (H+L) secondary antibody (Invitrogen, A-11055). Coverslips were mounted on slides with Fluoromount-G with DAPI (Invitrogen) and analyzed by confocal microscopy (Zeiss LSM 880). Frequencies of NET-producing cells were determined by counting at least 200 cells per condition. Each condition was evaluated in triplicate in a blinded manner by coding of samples. Neutrophils that released filamentous structures containing DNA and MPO were considered as NET-forming cells.

### Reactive Oxygen Species (ROS) Production

ROS production was measured using a luminol-based chemiluminescence assay, as previously described (Regli et al., 2018). Neutrophils were resuspended in an RPMI medium at 3 x 10^6^ c/ml and distributed into the wells of white opaque 96-well plates. Cells were infected with *L. (V.) panamensis* lines or clinical strains, and ROS production was evaluated immediately and 2 h after infection over 60-min periods in the absence or presence of 32 µg SbV/ml as MA. Luminol Sodium Salt (Carbosynth Limited) was added at a final concentration of 20 µg/ml, and luminescence induced by ROS was measured every 2.5 min for periods of 60 min using a plate reader (Chamaleon). Positive control for ROS production was induced with 100 ng/ml PMA.

### Neutrophil Membrane Activation Marker Staining

To evaluate the viability of neutrophils, cells were stained with the LIVE/DEAD TM Fixable Green Dead Cell Stain Kit (Life Technologies) for 15 min at 4°C and washed once with PBS. Neutrophils were distributed at 5x10^5^ cells per tube and stained for 20 min at 4°C with a negative cocktail of antihuman CD3/CD19/CD14 (PerCP); positive staining of neutrophils was performed with antihuman CD15 (APC) and activation markers as follows: antihuman CD66b, CD18, and CD62L (PE). Flow cytometry was performed using a Becton Dickinson Accuri C6 Flow Cytometer, and data were analyzed using FlowJo V10 (Tree Star). For each experimental sample, 100,000 events were acquired based on the neutrophils CD15+ gate.

### Data Analysis

The Mann–Whitney U test or unpaired t test was used to establish statistical differences between neutrophil activation induced by sensitive and resistant strains. To compare two related groups (the same sample of neutrophils in the absence or presence of drug), Wilcoxon matched-pairs signed rank test or paired t was used according to the parametric or nonparametric distribution of data. Statistical differences among groups for dsDNA release in supernatant were analyzed by Kruskal–Wallis followed by Dunn’s multiple comparison test. Two-way ANOVA was used to compare NET release assessed by microscopy in the absence or presence of drug. Analyses were performed with the GraphPad Prism 6 software (GraphPad Inc., San Diego, CA), and P values < 0.05 were considered significant.

## Results

### Infection of Neutrophils by Intrinsically Sensitive and SbV Resistant Clinical Strains of *L. (V.) panamensis* Differentially Elicit ROS Induction

**Figure 1:**
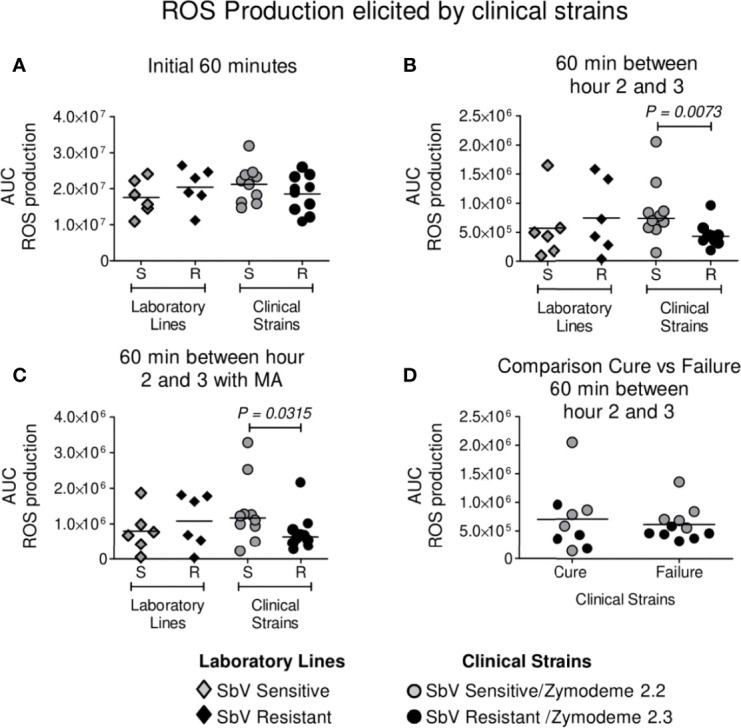
ROS production by neutrophils during infection with intrinsically SbV sensitive (S) (zymodeme 2.2) and tolerant/resistant (R) (zymodeme 2.3) clinical strains of *L. (V.) panamensis*. ROS production was evaluated over 60 min immediately after infection **(A)** and between hours 2 and 3 following infection in the absence **(B)** or presence of antimony **(C)**. Comparison of ROS induction by clinical strains in relation with treatment outcome (11 failures, 9 cures) of the corresponding patient during 60 min between hours 2 and 3 **(D)**. Neutrophils isolated from healthy donors were infected with sensitive (n = 10) and antimony-resistant (n = 10) clinical strains; susceptible and resistant laboratory lines were included in each experiment. ROS production was determined independently in neutrophils from three healthy donors. Data analysis was conducted using unpaired t test **(A)** or the Mann–Whitney **(B, C)** according to the parametric or nonparametric distribution of data. P value shown for comparisons presenting significant differences.

**Figure 2:**
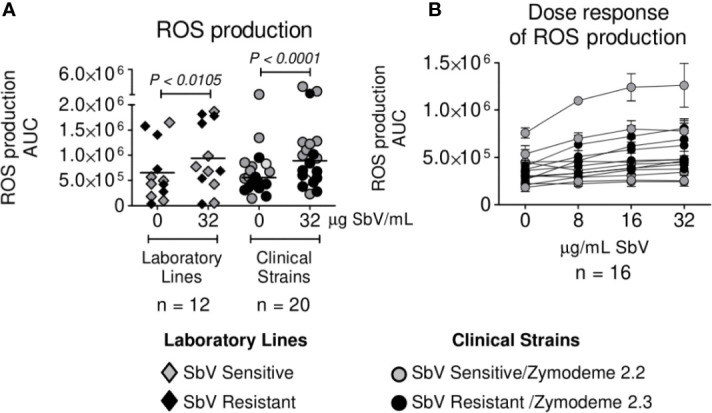
Antimony effect on ROS production by neutrophils infected with sensitive and antimony tolerant/resistant clinical strains of *L. (V.) panamensis*. Effect of SbV at Cmax: 32 ug SbV/ml on ROS production **(A)**. Dose response of ROS production by infected neutrophils over a range of clinically relevant concentrations of antimony **(B)**. ROS production is expressed as AUC. Data correspond to ROS production by neutrophils from three donors.

Neutrophils were infected with clinical strains of *L. (V.) panamensis* having intrinsic sensitivity (zymodeme 2.2) or resistance (zymodeme 2.3) to SbV over a period of 3 h. A trend toward a lower generation of ROS by neutrophils exposed to intrinsically SbV tolerant/resistant strains compared to drug-sensitive clinical strains was evident at 60 min (**Figure 1A**) and reached significance during the interval from 2 to 3 h of infection (**Figures 1B, C**), both in the presence of the C_max_ of SbV (32 µg/ml) or in the absence of drug (**Figures 1B, C**). An exploratory comparison of ROS induction by clinical strains in relation with the treatment outcome of the corresponding patient with MA did not reveal significant differences in ROS induction by clinical strains from patients who failed or responded to treatment (**Figure 1D**). This was ascertained in the absence of drug (at 2 to 3 h/AUC median: Failure 5.4 x 10^5^ *vs* Cure AUC 5.7 x 10^5^, P = 0.5 as well as at 60 min/AUC mean: Failure 2.0 x 10^7^ *vs* Cure 1.9 x 10^7^, P = 0.2962); and the presence of MA (at 2 to 3 h/AUC median: Failure AUC 0.7 x 10^6^ *vs* Cure AUC 1.0 x 10^6^, P = 0.3520). Independently of the susceptibility phenotype of the infecting parasites, exposure of infected neutrophils to MA at a concentration of 32 ug SbV/ml elicited a significant increase in ROS production compared to infected neutrophils cultured without drug (**Figure 2A**). The modulation of ROS production by MA exposure of neutrophils infected with *L. (V.) panamensis* was confirmed by the dose-dependent response to four clinically relevant concentrations of SbV (**Figure 2B**). These results were replicated using neutrophils obtained from three healthy donors.

### NET Formation by Neutrophils Is Similar Following Infection by SbV Sensitive and Resistant Clinical Strains of *L. (V.) panamensis*

**Figure 3:**
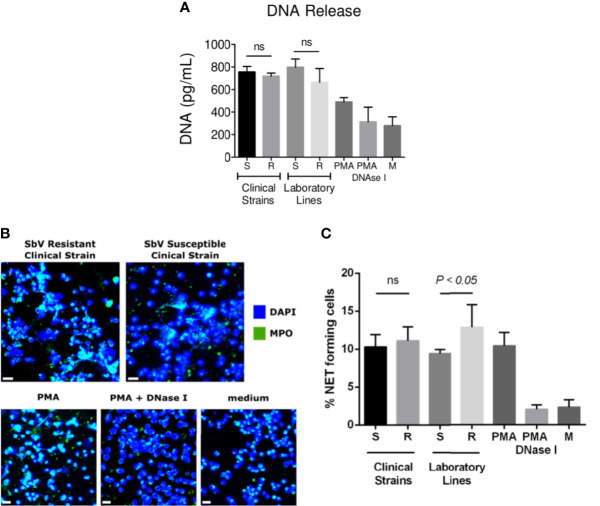
NET release by neutrophils infected with sensitive and antimony tolerant/resistant clinical strains of *L. (V.) panamensis.* Neutrophils were exposed to *L. (V.) p.* parasites of the indicated susceptibility. Four hours later, NET formation was evaluated. Levels of dsDNA released in the culture supernatant by fluorescent picogreen assay **(A)**. Confocal microscopy images of neutrophils seeded on poly-L-lysine wells and exposed to *L. (V.) p.* parasites. As controls, neutrophils were exposed to PMA, PMA + DNase I, or the medium alone. Cells were fixed and stained for human MPO (green) and DNA (blue). The scale bar represents 20 µm **(B)**. Frequency of NET-forming neutrophils assessed by confocal microscopy **(C)**. Data are the mean frequency of NET-producing neutrophils observed in three different neutrophil donors. M, medium; ns, not significant.

Neutrophils were infected during 4 h with sensitive or resistant clinical strains in the absence or presence of 32 µg SbV/ml during the last 2 h of culture. Release of DNA was analyzed as a surrogate of NETs at the end of 4 h. Neutrophils exposed to sensitive or resistant clinical strains of *L. (V.) panamensis* released similar concentrations of DNA into the culture supernatant (**Figure 3A**). Neutrophils infected with antimony-resistant and sensitive laboratory lines, which were included in each experiment with clinical strains, also showed no differences in DNA release (**Figure 3A**). As the picogreen assay may detect DNA release not specific to NETs, we further visualized *L. (V.) panamensis* induced NET formation by confocal microscopy. To this end, neutrophils were exposed to sensitive or resistant clinical strains, and formation of NETs was assessed by confocal immunofluorescent microscopy using DAPI to detect filamentous DNA structures and an antibody against MPO to detect the MPO associated with these structures. As a positive control, neutrophils were exposed to PMA, and as negative controls to PMA and DNAse, or the medium only. We observed that all the clinical strains analyzed induced NET formation independently of their intrinsic or natural susceptibility phenotype (**Figure 3B**). Neutrophils infected with laboratory-adapted lines also produced NETs, in particular, when exposed to the SbV-resistant laboratory strain as previously observed (Regli et al., 2018) (**Figure 3C**).

### NET Release by Infection With Clinical Strains of *L. (V.) panamensis* Is Modulated by SbV as MA

**Figure 4:**
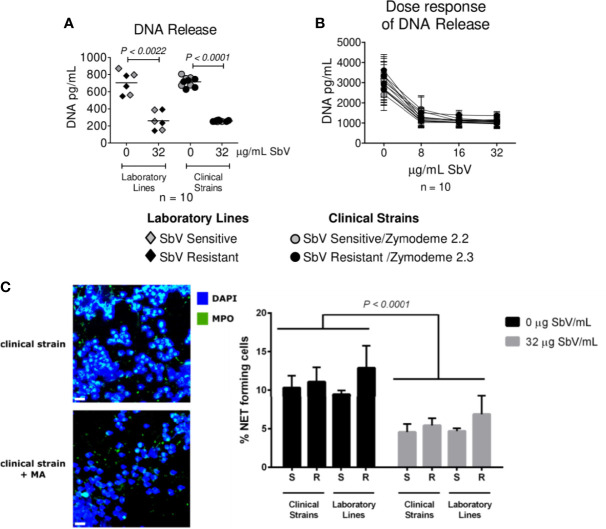
Antimony effect on NET release by neutrophils infected with sensitive and antimony tolerant/resistant clinical strains of *L. (V.) panamensis*. Effect of SbV at Cmax: 32 ug SbV/ml on DNA release **(A)**. Dose response effect on DNA release **(B)**. Effect of SbV exposure on NET formation evaluated with confocal microscopy. On the left, representative confocal images of neutrophils exposed to the clinical strain of *L. (V.) p.* in the absence or presence of SbV. On the right, quantification of NET-forming neutrophils **(C)**. Data are the mean frequency of NET-producing neutrophils observed in three different neutrophil donors.

Next, we evaluated whether MA exposure would alter NET formation. Neutrophils were exposed to sensitive or resistant clinical strains, in the absence or presence of 32 µg SbV/ml or a range of relevant drug concentrations, during the last 2 h of culture. Picogreen assay revealed a diminished DNA release in infected neutrophils cultured with drug compared to those cultured without drug, both at Cmax and over a range of clinically relevant concentrations (**Figures 4A, B**). These results were corroborated by the analysis of NET formation by confocal microscopy, where all clinical and laboratory lines assessed induced a lower amount of NETs upon MA exposure (**Figure 4C**). Although MA exposure resulted in reduced NET release, the response was not dose-dependent, as reduction of NETs observed over the concentration range of 8 to 32 µg SbV/ml was similar (**Figure 4B**). Viability of neutrophils under the conditions and over the time course of assays of ROS and NET production was evaluated by MTT assay and shown to be >90%.

### Neutrophil Surface Activation Marker Expression Is Modulated by Infection With SbV Sensitive or Resistant Strains of *L. (V.) panamensis*, and Exposure to MA

**Figure 5:**
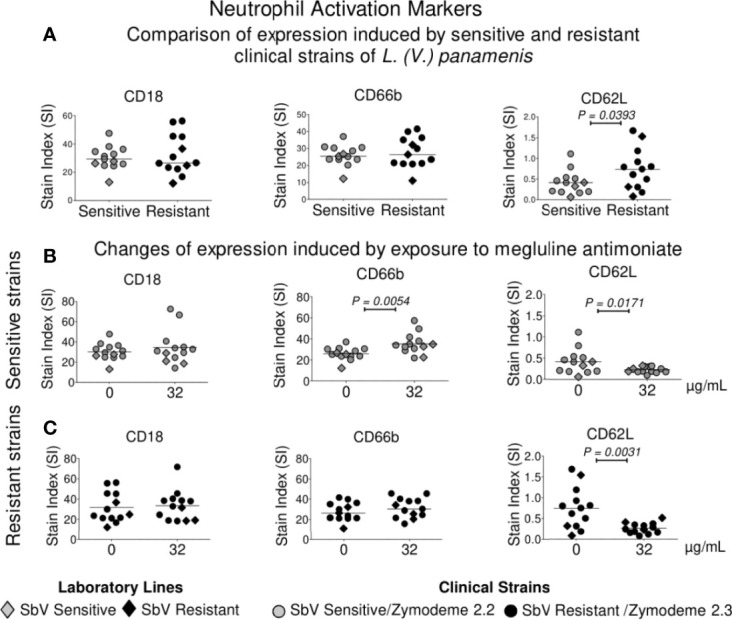
Modulation of activation markers in neutrophils following infection with sensitive and resistant strains of *L. (V.) panamensis* and antimony exposure. Expressions of CD18, CD66b, and CD62L were compared in neutrophils infected with intrinsically SbV sensitive and resistant clinical strains of *L. (V.) panamensis* **(A)**, and after exposure to 32 µg SbV/ml of neutrophils infected with sensitive (n = 10) **(B)** and resistant strains (n = 10) **(C)**. Orange circles represent sensitive and resistant laboratory lines of *L. (V.) panamensis*. Each point corresponds to the mean or median of marker expression on neutrophils from three healthy donors. Statistical significance was defined using paired t test or Wilcoxon test according to the distribution of the data.

Neutrophils infected with SbV sensitive and resistant clinical strains of *L. (V.) panamensis*, in the absence of drug exposure, exhibited similar expression of CD18 and CD66b activation markers, whereas the expression of CD62L, a marker that is lost during cell homing and diapedesis into peripheral tissues, was significantly higher in neutrophils infected with SbV resistant strains (**Figure 5A**). Exposure of infected neutrophils to antimonial drug resulted in a higher expression of the activation marker CD66b, reaching significance for neutrophils infected with SbV sensitive strains (**Figure 5B**), and significantly diminished the expression of CD62L in neutrophils infected with drug sensitive as well as resistant strains (**Figures 5B, C**).

## Discussion

The results of this study show that infection of neutrophils with phenotypically/genotypically distinguishable clinical strains of *L. (V.) panamensis* that are intrinsically sensitive or resistant to SbV differentially modulates neutrophil activation. These findings obtained with clinical strains extend prior observations based on laboratory-derived lines (Regli et al., 2018) by substantiating that parasite adaptations that have occurred over time during the natural cycle of transmission contribute to or confer tolerance or resistance to antileishmanial drugs and are associated with modified host cell responses to infection. Naturally occurring adaptations of *Leishmania* populations causing cutaneous leishmaniasis that enhance intracellular survival despite antimicrobial defense mechanisms, can also contribute to the effect of drug exposure on host cells as well as the parasite responses to antileishmanial drugs. This phenomenon has been previously evidenced in the modulation of drug transport by SbV in infected host macrophages (Gomez et al., 2014) and as cross-tolerance to nitric oxide (NO) reported among strains of *L. (V.) braziliensis* from a group of patients who failed antimonial treatment (Souza et al., 2010). However, parasite susceptibility to SbV was not evaluated in the latter study. Similarly, four clinical strains of *Leishmania infantum* isolated from patients who relapsed following treatment with MA drug presented tolerance to NO and to trivalent antimony (Sb^III^). Modulation of neutrophil responses by our cohort of 20 clinical strains of *L. (V.) panamensis* was associated with intrinsic drug susceptibility phenotype for SbV, though not with outcome of treatment, which is multifactorial. Adherence to treatment, age-related pharmacokinetics, chronicity and location of lesions, and concurrent conditions influence and can confound the therapeutic response (Castro et al., 2017).

The intrinsic nature of the difference in susceptibility to SbV of the *L. (V.) panamensis* populations (zymodeme 2.2 and 2.3) represented by the clinical strains included in this study is supported by their sympatric prevalence and distribution throughout the Pacific Coast region of Colombia (Saravia et al., 1998). Furthermore, classification of the represented populations within distinct zymodemes was established during the 1990s and reported in 1998 (Saravia et al., 1998), 15 years before our large-scale drug susceptibility evaluation revealed their disparate susceptibility phenotype for antimonial drugs (Fernandez et al., 2014). Although the bases of the tolerant/resistant phenotype are not known, the results of this study support the participation of the elicited response of host neutrophils to the distinct parasite populations and to MA, as well as intrinsic parasite susceptibility to SbV, underscoring the interdependency of the host–parasite–drug interaction.

The observed induction of lower ROS production by SbV resistant clinical strains is potentially a manifestation of an adaptive phenotype that would diminish the effectiveness of this neutrophil defense mechanism during intracellular infection and contribute to tolerance to Sb. Notably the induction of ROS by SbV as MA, which has been previously documented for macrophages infected with *Leishmania donovani* (Mandal et al., 2007), did not override the diminished ROS induction by SbV resistant clinical strains of *L. (V.) panamensis*. In contrast, ROS induction was not modulated by infection with the SbV resistant laboratory derived line of *L. (V.) panamensis*, consistent with the intrinsic basis of the diminished ROS induction by the SbV resistant clinical strains. Although resistance mechanisms experimentally selected by long term, incremental drug pressure have been informative of factors involved in intracellular distribution, metabolism, and elimination of antileishmanials, they have not modeled natural resistance (Goyeneche-Patino et al., 2008). Hence, the mechanism(s) underlying tolerance to SbV in these clinical strains offers a window on the pathways to intracellular survival of *Leishmania* in the presence of this antileishmanial as well as in the absence of drug.

The participation of modulated host cell responses in treatment outcome was not discernable in the limited number of clinical strains from patients whose treatment outcome was available for inclusion in this analysis. The multiplicity of host, parasite, and herein demonstrable parasite-mediated host responses confound and challenge the association of specific factors with therapeutic outcome. Nevertheless, the evidence of down-modulation of ROS production and increased expression of the surface activation marker and L-selectin associated with homing and migration to inflammatory sites in neutrophils infected with clinical strains that are intrinsically resistant is consistent with adaptations that favor the establishment of infection. The lower ROS induction by SbV tolerant/resistant clinical strains compared to sensitive strains extends prior findings with laboratory-derived lines to epidemiologically and clinically relevant parasite populations. The nature and mechanism of intrinsic tolerance/resistance are likely to be distinct from mechanisms derived by experimental selection based on direct and specific drug pressure and motivate further examination of neutrophil responses to phenotypically distinctive natural populations of *Leishmania* infecting patients.

SbV as meglumine antimoniate modulated some of the principal antimicrobial mechanisms of neutrophils, augmenting ROS production and reducing NET formation. These contrasting effects on neutrophil activation were consistently manifest independently of the susceptibility phenotype, whether laboratory derived or intrinsic to naturally circulating populations of *L. (V.) panamensis*. Several mechanisms may induce NET release that may be ROS-dependent (suicidal NETosis) (Fuchs et al., 2007) or ROS-independent (vital NETosis) (Pilsczek et al., 2010; Rochael et al., 2015). NET formation was induced in response to all the clinical *L. (V.) panamensis* strains tested irrespective of their sensitivity phenotype. Here, despite an increase in ROS production observed in the presence of parasites and MA, we observed decreased release of NETs following infection of drug-exposed neutrophils of both sensitive and resistant *L. (V.) panamensis* strains. It has been shown that the requirement of ROS for NET formation depends on the stimulus (Parker et al., 2012); in this regard, *L. amazonensis* has been reported to induce both the formation of ROS-dependent and ROS-independent NET release. As the increased ROS production induced by SbV did not correlate with increased NET release, our data suggest that the negative impact of MA on NET induction is ROS-independent.

The *in vitro* antimony concentrations, 8 to 32 µg SbV/ml, used in this study are achieved in plasma during treatment (Cruz et al., 2007). However, the implications of these drug-mediated effects on neutrophil defense mechanisms in relation with therapeutic response will require the evaluation of neutrophil responses of patients during treatment. The differential neutrophil activation observed following infection with clinical strains of *L. (V.) panamensis* that are intrinsically resistant to SbV illustrates the interplay of naturally occurring parasite adaptations and the elicited host cell response in antileishmanial drug susceptibility and resistance. This finding together with the evidence that antimonial drug also modulates neutrophil antimicrobial mechanisms enlightens approaches to optimizing therapeutic strategies.

Healing of CL is dependent upon the control of pro- and anti-inflammatory host defense mechanisms (Lakhal-Naouar et al., 2015). Consequently, antileishmanial drugs alone are often insufficient to clinically resolve disease even in immuno-competent individuals. Differential effects of intrinsically SbV tolerant/resistant *L. (V.) panamensis* on neutrophil activation and antimicrobial responses such as decreased ROS may influence therapeutic response to this drug. Modulation of host responses that impede lesion resolution and mobilization of regulatory mechanisms that promote healing may be achievable through co-adjuvant immunotherapeutic approaches to the treatment of cutaneous leishmaniasis (Ehrlich et al., 2017; Thacker et al., 2020).

## Data Availability Statement

The raw data supporting the conclusions of this article will be made available by the authors, without undue reservation.

## Ethics Statement

The studies involving human participants were reviewed and approved by the Institutional Ethical Review Board of Centro Internacional de Entrenamiento e Investigaciones Médicas (CIDEIM) and by the Ethical Committee of the Canton of Vaud and the Swiss Confederation (CER-VD 2017-00182). The patients/participants provided their written informed consent to participate in this study.

## Author Contributions

NGS, FT-C, OF, LR, and MD-V contributed to the design and interpretation of the experiments. LR, OF, and MD-V performed the experiments and analyzed the data. NGS, OF, and LR wrote the manuscript. FT-C and MD-V contributed to and critically reviewed the manuscript. All authors contributed to the article and approved the submitted version.

## Funding

This work was supported by the SPIRIT Swiss Programme for International Research by Scientific Investigation Teams, number IZSTZO_1190140 to FT-C and NGS, and in part by the Swiss National Science Foundation grant number 310030_184751 to FT-C and by the Global Infectious Research Training Program of the Fogarty International Center of the U.S. National Institutes of Health under award number SD43TW006589 and NIH/NIAID Tropical Medicine Research Centers (TMRC) grant U19AI129910 to NGS.

## Conflict of Interest

The authors declare that the research was conducted in the absence of any commercial or financial relationships that could be construed as a potential conflict of interest.

## Publisher’s Note

All claims expressed in this article are solely those of the authors and do not necessarily represent those of their affiliated organizations, or those of the publisher, the editors and the reviewers. Any product that may be evaluated in this article, or claim that may be made by its manufacturer, is not guaranteed or endorsed by the publisher.
